# Association of Osteoporosis Self-Assessment Tool for Asians (OSTA) Score with Clinical Presentation and Expenditure in Hospitalized Trauma Patients with Femoral Fractures

**DOI:** 10.3390/ijerph13100995

**Published:** 2016-10-10

**Authors:** Chien-Chang Chen, Cheng-Shyuan Rau, Shao-Chun Wu, Pao-Jen Kuo, Yi-Chun Chen, Hsiao-Yun Hsieh, Ching-Hua Hsieh

**Affiliations:** 1 Department of Plastic Surgery, Kaohsiung Chang Gung Memorial Hospital, Kaohsiung 83301, Taiwan; kschen@cgmh.org.tw (C.-C.C.); bow110470@gmail.com (P.-J.K.); libe320@yahoo.com.tw (Y.-C.C.); sylvia19870714@hotmail.com (H.-Y.H.); 2 Chang Gung University College of Medicine, Taoyuan City 33302, Taiwan; ersh2127@cloud.cgmh.org.tw (C.-S.R.); shaochunwu@gmail.com (S.-C.W.); 3 Department of Neurosurgery, Kaohsiung Chang Gung Memorial Hospital, Kaohsiung 83301, Taiwan; 4 Department of Anesthesiology, Kaohsiung Chang Gung Memorial Hospital, Kaohsiung 83301, Taiwan

**Keywords:** osteoporosis, Osteoporosis Self-Assessment Tool for Asians (OSTA), trauma, femoral fracture, injury severity score (ISS), length of stay (LOS), propensity-score matching

## Abstract

*Background*: A cross-sectional study to investigate the association of Osteoporosis Self-Assessment Tool for Asians (OSTA) score with clinical presentation and expenditure of hospitalized adult trauma patients with femoral fractures. *Methods*: According to the data retrieved from the Trauma Registry System between 1 January 2009 and 31 December 2015, a total of 2086 patients aged ≥40 years and hospitalized for treatment of traumatic femoral bone fracture were categorized as high-risk patients (OSTA < −4, *n* = 814), medium-risk patients (−1 ≥ OSTA ≥ −4, *n* = 634), and low-risk patients (OSTA > −1, *n* = 638). Two-sided Pearson’s, chi-squared, or Fisher’s exact tests were used to compare categorical data. Unpaired Student’s *t*-test and Mann-Whitney *U*-test were used to analyze normally and non-normally distributed continuous data, respectively. Propensity-score matching in a 1:1 ratio was performed using Number Crunching Statistical Software (NCSS) software (NCSS 10; NCSS Statistical Software, Kaysville, UT, USA), with adjusted covariates including mechanism and Glasgow Coma Scale (GCS); injuries were assessed based on the Abbreviated Injury Scale (AIS), and Injury Severity Score (ISS) was used to evaluate the effect of OSTA-related grouping on a patient’s outcome. *Results*: High-risk and medium-risk patients were predominantly female, presented with significantly older age and higher incidences of co-morbidity, and were injured in a fall accident more frequently than low-risk patients. High-risk patients and medium-risk patients had a different pattern of femoral fracture and a significantly lower ISS. Although high-risk and medium-risk patients had significantly shorter lengths hospital of stay (LOS) and less total expenditure than low-risk patients did, similar results were not found in the selected propensity score-matched patients, implying that the difference may be attributed to the associated injury severity of the patients with femoral fracture. However, the charge of surgery is significantly lower in high-risk and medium-risk patients than in low-risk patients, regardless of the total population or the selected propensity score-matched patients. This lower charge of surgery may be attributed to a less aggressive surgery applied for older patients with high or medium risk of osteoporosis. *Conclusions*: This study of hospitalized trauma patients with femoral fracture according to OSTA risk classification revealed that high-risk and medium-risk patients had significantly higher odds of sustaining injury in a fall accident than low-risk patients; they also present a different pattern of femoral bone fracture as well as a significantly lower ISS, shorter hospital LOS, and less total expenditure. In addition, the significantly lower charge of surgery in high-risk and medium-risk patients than in low-risk patients may be because of the preference of orthopedists for less aggressive surgery in dealing with older patients with osteoporotic femoral bone fracture.

## 1. Background

With the proportion of elderly population increasing, osteoporosis has rapidly become a growing global health issue because of the exponentially increasing prevalence and associated morbidity and costs [1]. Burge et al. estimated that the occurrence of osteoporotic fractures will increase to 3.5 million in 2025 from the 2 million reported in 2005 [2]. Fractures of the proximal femur are greatly exacerbated by osteoporosis and present as the most typical and serious complications of osteoporosis [3,4,5,6]. It has been estimated that, in women, for every one standard deviation decrease in bone mineral density (BMD) below the mean age, there is an age-adjusted relative increase of 1.4 in the risk of femoral neck fracture [7]. In addition, femoral fracture is associated with considerable morbidity [8,9]. Twenty percent of patients who have a hip fracture die within 12 months and only half of the patients regain the mobile independence they had before the occurrence of hip fracture. Moreover, almost half of those who can walk without assistance are no longer able to do so and half of those who survive the accident are no longer able to live independently [1,10,11]. After a hip fracture, the proportion of patients living in nursing homes increased from 15% to 30% [10]. Thus, a remarkable increase in the burden on the healthcare system is expected for those patients with osteoporotic femoral bone fracture.

In addition to advanced age, low body weight is also strongly associated with low BMD, as well as with an increased risk of bony fracture [12,13,14,15]. Although the measurement of BMD by dual energy X-ray absorptiometry remains the gold standard method for diagnosing osteoporosis [13], the World Health Organization (WHO) developed the Osteoporosis Self-Assessment Tool for Asians (OSTA) score based simply on age and body weight to identify the women at risk for osteoporosis [16]. The OSTA is calculated using the following formula: (body weight (kg) − age (year)) × 0.2, with the decimal digits being disregarded [16]. Patients are stratified into patients at low (OSTA > −1), medium (−1 ≥ OSTA ≥ −4), and high risk (OSTA < −4) of sustaining osteoporosis [11,17]. The OSTA has been validated as an effective, feasible screening tool to identify patients with low BMD and the risk of sustaining osteoporosis [16,18,19,20,21,22,23,24]. The risk of osteoporosis in the high-, medium- and low-risk categories was found to be 61%, 15% and 3%, respectively [16]. A significant positive correlation was found between the OSTA index and T-scores of BMD measured by dual energy X-ray absorptiometry at the femoral neck [24,25]. The probability of a patient with an OSTA score < −4 having osteoporosis is 53.8% [23]. The probability of a patient with an OSTA score > −4 not having osteoporosis is 99.3% [23]. Using an OSTA threshold of ≤ −1 allows for identification of osteoporotic subjects with femoral neck BMD T-scores ≤ −2.5, with a specificity of 97.0% and sensitivity of 43.1% in postmenopausal Chinese women aged 45–59 years old [24]. Although osteoporosis is less prevalent in men than women, 30% of hip fractures occur in men [26] and comprise around 25% of the total cost burden [2]. Similar supporting correlations have also been reported from OSTA studies in men [17,18,25,27]. In addition, studies on OSTA report strong correlations not only for the Taiwanese population [28,29] but also for populations of the Philippines, Japan, Korea, Malaysia, Thailand, Singapore, and China [16,19,20,21,22,23,24,30].

In Taiwan, the prevalence of osteoporosis was 41.3% in a cohort study of 12,175 Taiwanese postmenopausal women [31] and 38.3% in Taiwanese women aged ≥ 50 years old [32]. In addition, with the age-specific incidence rates of hip fractures increasing exponentially after 65 years of age in both sexes, the Taiwanese population had among the highest hip fracture rates in the world [33,34]. From 1996 to 2002, the overall incidence of hip fractures in Taiwan showed a significant 30% increase from 49.6 to 64.4 hip fractures per 10,000 people annually [35]. However, currently, little is known regarding the impact of osteoporosis on the pattern of fracture of the femoral bone and in-hospital outcome of trauma patients with femoral fracture. Therefore, we aimed to investigate the association between OSTA and clinical presentation and expenditure of hospitalized adult trauma patients with femoral fracture in a Level I trauma center in southern Taiwan.

## 2. Methods

### 2.1. Study Design

**Figure 1:**
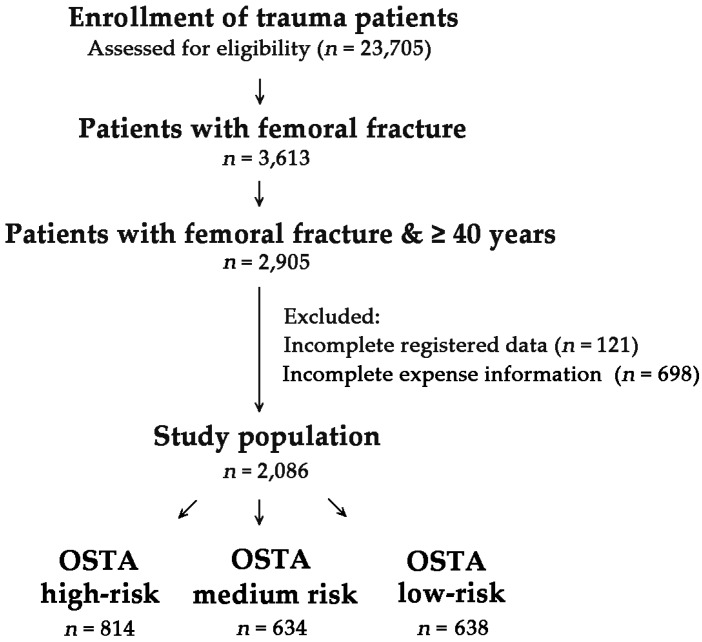
Flow chart of the patients selected for study.

This retrospective study reviewed data of all 23,705 patients enrolled in the Trauma Registry System from 1 January 2009 to 31 December 2015 (Figure 1). The patients who were ≥40 years of age and hospitalized for treatment of traumatic femoral bone fracture were included in the study. The OSTA was calculated based on age and body weight by using the following formula: (body weight (kg) − age (year)) × 0.2 [16]. Patients with an incomplete OSTA value or those who were lacking information pertaining to hospital expenditure were excluded. This inclusion group consisted of 2086 patients with (1) 814 high-risk patients (OSTA < −4); (2) 634 medium-risk patients (−1 ≥ OSTA ≥ −4), and (3) 638 low-risk patients (OSTA > −1). Detailed patient information retrieved from the Trauma Registry System of our institution included the following: age; sex; co-morbidities such as diabetes mellitus (DM), hypertension (HTN), coronary artery disease (CAD), congestive heart failure (CHF), and cerebral vascular accident (CVA); trauma mechanism; blood alcohol concentration (BAC) level; initial Glasgow Coma Scale (GCS) score in the emergency department (ED); severity score of Abbreviated Injury Scale (AIS) in each body region; Injury Severity Score (ISS); rates of associated injuries; femur bone-related surgery; length of stay (LOS) in hospital; LOS in intensive care unit (ICU); in-hospital mortality; and total expenditure, which included charge of the surgery (fees for surgery and surgical supplies), charge of examination (fees for physical examination, hematology testing, radiography examination, pathological examination, electrocardiography examination, echo, endoscopy, electromyography, and cardiac catheterization, and electroencephalography monitoring), charge of pharmaceuticals (fees for medical services, medicines, and narcotic drugs), and other charges (fees for registration, administration, wards, nursing, blood/plasma testing, hemodialysis, anesthesia, rehabilitation-treatment, special material charges, and personal expenses), which was expressed in U.S. dollars as charge per patient. The fracture sites were recorded according to their location, which were divided into proximal femur, which including type A (trochanteric); type B (neck); and type C (head), shaft and distal femur according to the Arbeitsgemeinschaft für Osteosynthesefragen (AO) classification [36]. Patients with a BAC level of ≥50 mg/dL at the time of arrival at ED were considered intoxicated. The ISS is expressed as the median and interquartile range (IQR, Q1–Q3). The data collected were compared using IBM SPSS Statistics for Windows, version 20.0 (IBM Corp., Armonk, NY, USA). Odds ratios (ORs) of the associated conditions and injuries of the patients were calculated with 95% confidence intervals (CIs). Pearson, chi-squared test or two-sided Fisher’s exact test were used to compare categorical data. The continuous data were expressed as mean ± standard deviation. Unpaired Student’s *t*-test and Mann-Whitney *U*-test were used to analyze normally and non-normally distributed continuous data, respectively. To minimize the confounding effects of non-randomized assignment in the assessment of OSTA grouping on outcome, which included in-hospital LOS, ICU admission, and medical expenses, propensity scores were calculated using Number Crunching Statistical Software (NCSS) software (NCSS 10; NCSS Statistical Software, Kaysville, UT, USA). These were calculated with the following covariates: mechanism; GCS, injuries based on AIS, and ISS. Age, sex, and co-morbidities as inherent characteristics of the OSTA were not adjusted as a covariate in the calculation of propensity score. A separate 1:1 matched set of comparable study populations for the high-risk vs. low-risk and the medium-risk vs. low-risk patients was created by the greedy method after adjustment of these confounding factors. Binary logistic regression was performed to assess the effect of OSTA-related grouping on patient outcomes. *p*-values < 0.05 were considered statistically significant.

### 2.2. Ethical Statement

This study was approved by the Institutional Review Board (IRB) of the Chang Gung Memorial Hospital (approval number 201600518B0). According to IRB regulations, informed consent was waived.

## 3. Results

### 3.1. Injury Characteristics and Severity of the Patients with Femoral Fracture

As shown in Table 1, the mean age of high-risk patients and medium-risk patients was higher than that of low-risk patients. Significantly greater female predominance was noted both in high-risk patients and medium-risk patients than in low-risk patients. Both high-risk and medium-risk patients had higher odds of HTN, CAD, and CHF than low-risk patients did. In addition, medium-risk patients, but not high-risk patients, had higher odds of DM and CVA than low-risk patients did. More high-risk patients (90.0%) and medium-risk patients (74.8%) were injured in a fall accident than low-risk patients (53.9%); in contrast, more low-risk patients sustained injuries in a motor vehicle or motorcycle accident, as a pedestrian, and from being struck by/against objects. Significantly fewer high-risk patients, but not medium-risk patients, were injured in bicycle accidents than low-risk patients. A significantly lower incidence of positive BAC was found in the high-risk and medium-risk patients than low-risk patients. GCS scores were not significantly different both in high-risk patients and medium-risk patients compared to low-risk patients. Analysis of AIS, under the criteria of AIS ≥ 3, revealed that high-risk patients and medium-risk patients had sustained significantly lower rates of head and neck, face, thoracic, and abdominal injuries than low-risk patients had. For these patients with femoral bone fracture, the associated injuries with AIS ≥ 3 in body regions other than the extremities accounted for generally less than 10%; however, 11.3% of head and neck injuries were associated with being in a low-risk patient group. In addition, a significantly lower ISS was found in high-risk patients and medium-risk patients than in low-risk patients, notwithstanding the difference of ISS between these groups was less than one point. However, when stratified by ISS (<16, 16–24 or ≥25), fewer high-risk patients and medium-risk patients had an ISS of 16–24 or an ISS ≥ 25 than low-risk patients did. In contrast, more high-risk patients and medium-risk patients had an ISS of <16 than low-risk patients.

### 3.2. Outcome of the Patients with Femoral Fracture

The high-risk and medium-risk patients did not show significantly different in-hospital mortality compared to low-risk patients (Table 1). However, compared to low-risk patients, high-risk patients had significantly shorter hospital LOS (9.9 days vs. 11.1 days, respectively; *p* = 0.010), and medium-risk patients had significantly shorter hospital LOS (9.9 days vs. 11.1 days, respectively; *p* = 0.028), a lower proportion of patients admitted to the ICU (6.6% vs. 10.0%, respectively; *p* = 0.028), and a shorter ICU LOS (5.4 days vs. 8.6 days, respectively; *p* = 0.016). In addition, compared to low-risk patients, high-risk patients had significantly less total expenditure (U.S. $3,459 ± 2813 vs. U.S. $4,451 ± 5014, or 28.9% lower, *p* < 0.001) and a lower surgery charge (U.S. $539 ± 209 vs. U.S. $785 ± 655, *p* < 0.001), while medium-risk patients had significantly less total expenditure (US $3,654 ± 3195 vs. U.S. $4,451 ± 5014, or 22.8% lower, *p* < 0.001), surgery charges (U.S. $585 ± 343 vs. U.S. $785 ± 655, *p* < 0.001), and pharmaceutical charges (U.S. $155 ± 290 vs. U.S. $232 ± 721, *p* < 0.001).

**Figure 2:**
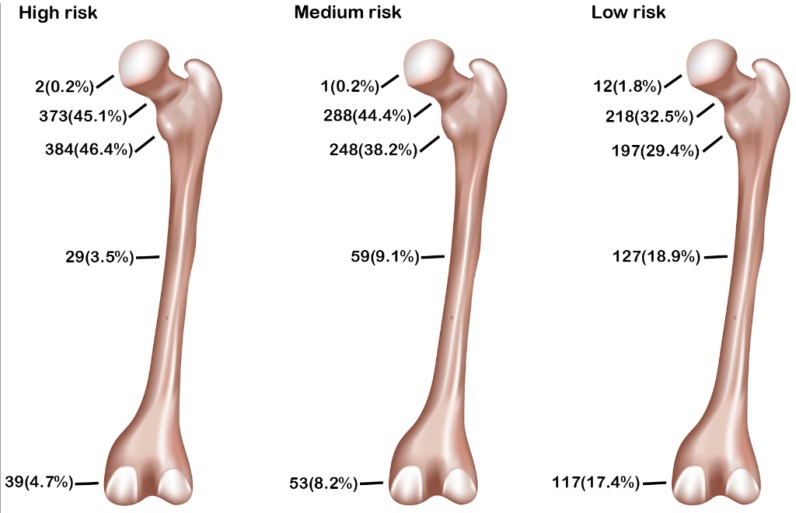
Femoral fracture sites of high-, medium-, and low-risk patients according to OSTA score.

**T**he fracture sites located in the trochanteric and neck region of femur bone comprised the majority of the fracture sites (Figure 2). The high-risk patients and medium-risk patients had greater odds of trochanteric fracture (2.1 and 1.5, respectively), as well as greater odds of femoral neck fracture (1.7 and 1.7, respectively), than low-risk patients (Table 2). In contrast, fewer high-risk patients and medium-risk patients had a femoral head, shaft, and distal femoral fracture than low-risk patients did. The in-hospital mortality was low for the patients with femoral fracture (Table 1), regardless of whether patients were at a high risk (*n* = 9, 1.1%), medium risk (*n* = 8, 1.3%), or low risk (*n* = 9, 1.4%); no significant difference was found among these groups of patients.

### 3.3. Outcome of Propensity-Score Matched Patients with Femoral Fracture

To minimize the confounding effects of the mechanism of injury, GCS, injuries based on AIS, and ISS related to OSTA effect on outcome, a separate set of propensity score-matched comparable study populations for high- and medium-risk vs. low-risk patients, respectively, was created for comparison. After propensity score matching, outcome was compared in the 408 well-balanced pairs of high-risk and low-risk patients (Table 3), 477 well-balanced pairs of medium-risk and low-risk patients (Table 4), and 545 well-balanced pairs of high-risk and medium-risk patients (Table 5). In these pairs of propensity score-matched patients, there was no significant difference in mechanism, GCS, injuries based on AIS, and ISS. The high-risk and medium-risk patients did not differ significantly in terms of the hospital LOS or medical expenses, implying that the aforementioned difference in hospital LOS and medical expenses observed between high-risk and low-risk patients as well as between medium-risk and low risk patients may be attributed to the associated injury severity of the patients with femoral fracture. Furthermore, the logistic regression analysis revealed there was no significant difference in the incidence of ICU admission. However, in the propensity score–matched patient population, high- and medium-risk patients still had a lower charge of surgery than low-risk patients did. The comparison of propensity-score matched high-risk vs. medium-risk patients revealed there was no significant difference of hospital LOS, incidence of ICU admission, and medical expenses, which including charge of surgery, charge of examination, and charge of pharmaceutical. Regarding femur-related surgeries, of 408 matched high-risk and low-risk patients, 248 surgeries had been performed in 246 high-risk patients (average 1.01 surgeries/patient) and 253 surgeries had been performed in 249 low-risk patients (average 1.02 surgeries/patient); of 477 matched medium- and low-risk patients, 298 surgeries had been performed in 297 high-risk patients (average 1.00 surgery/patient) and 291 surgeries had been performed in 287 low-risk patients (average 1.01 surgery/patient). No significant difference was found in the number of times the surgery was performed between high-risk or medium-risk patients with low-risk patients.

## 4. Discussion

This study compared the clinical outcome of patients hospitalized for femoral fracture according to the OSTA classification of associated risk of osteoporosis. In this study, high- and medium-risk patients were predominantly female and presented with significantly older age, higher incidences of co-morbidity, and were injured in a fall accident more frequently than low-risk patients. Compared to low-risk patients, high- and medium-risk patients had a different pattern of femoral fracture and a significantly lower ISS. Although high- and medium-risk patients had significantly shorter hospital LOS and less total expenditure than low-risk patients did, similar results were not found in the selected propensity-score-matched patients, implying that the difference may be attributed to the associated injury severity of the patients with femoral fracture. However, the charge of surgery is significantly lower in high-and medium-risk patients than in low-risk patients, regardless whether it is among the total population or the selected propensity score-matched patients.

In older age, proximal femoral fractures are mainly caused by a fall on the greater trochanter, not necessarily with high energy [3]. Some clinicians consider it sufficient to diagnose an osteoporotic fracture in adults aged above 50 who are in a condition from which an impact would not ordinarily cause a fracture (such as a fall from standing height) [15], regardless of the patient’s BMD [37]. In this study, as the high- and medium-risk patients were older than the low-risk patients and had greater odds of being injured in a fall accident than low-risk patients (7.7 and 2.5 times, respectively), it is not surprising to find that the high- and medium-risk patients had a different pattern of femoral fracture compared to the low-risk patients. The shape and structure of the femur is a vital structure in the determination of a person’s risk of fracture at the site around the hip [38]. In a fall accident, the force impacts directly onto the posterolateral aspect of the greater trochanter, making the femoral neck particularly vulnerable to fractures [39]. In a study of osteoporotic patients, a longer hip axis length (i.e., the distance from greater trochanter to inner pelvic brim) was found to create a greater bending moment in the femoral neck in a fall and was associated with an increased risk of trochanteric fractures (OR = 1.6; 95% CI: 1.0–2.4) and femoral neck (OR = 1.9; 95% CI: 1.3–3.0), when the greater trochanter made contact with the floor [40].

In this study, the average total inpatient charges of all patients was around U.S. $3,500–4500. The direct per-patient charges of hip fractures were estimated to be U.S. $7,000 and U.S. $12,890, respectively, for hospital care in different studies reported in 1997 [11] and 2002 [41]. In 2009, the direct in-hospital medical charges associated with hip fractures were U.S. $23,280 for patients 50–64 years of age and U.S. $11,925 for patients ≥65 years of age [42]. However, the comparison between this present study and other studies seemed to be unsuitable because of differences in the population composition, health care system, and insurance policy. It would be more suitable to demonstrate that the high-risk patients and medium-risk patients had a 28.9% and 22.8% lower charge in total expenditure than low-risk patients. As reported in the literature, osteoporotic fractures and the associated charges are expected to increase markedly as populations age [11]. In addition, 3–5 times greater morbidity related to osteoporotic hip fractures has been reported for both sexes at age ≥50 years [5]. All of the aforementioned conditions may imply a higher total expenditure of the high-risk and medium-risk patients who are usually older than the low-risk patients. However, in this study, a less total expenditure found in the high-risk and medium-risk patients than low-risk patients, seemingly in contrast to the expected findings. Notably, a significantly lower ISS was found in high-risk patients and medium-risk patients than in low-risk patients in this study, and there was no difference in total expenditure between the selected propensity score matched patients, indicating that an associated lower ISS may be one reason for the total expenditure being lower in the high-risk and medium-risk patients. The lower ISS may also result in a shorter hospital LOS, which is expected to be associated with less total expenditure.

Achieving stable fixation in osteoporotic fracture is difficult and tends to be complicated with insufficient pull-out strength of implants and the reduction of the bone regenerative capacity [43,44]. Therefore, failure of fixation is a common problem in the treatment of osteoporotic femoral fractures [45], especially in the treatment of those that are unstable and trochanteric [46]. In older osteoporotic patients, the fracture healing response was significantly slower [47]. In this study, we found no significant difference in the times of surgeries among the high-risk, medium-risk, or low-risk patients; however, the charge of surgery was significantly lower in high-risk and medium-risk patients than in low-risk patients, regardless whether it was among the total population or the selected propensity score-matched patients. From our observations, the lower charge of surgery in high-risk and medium-risk patients than that in low-risk patients may be attributed to the extent at which the orthopedists tend to manage the older high-risk patients with a less aggressive surgery such as traction or nailing but not plating or total hip replacement. However, in this retrospective study, the type of indication was not known, and this prevented further analysis. In addition, the price of those expensive plates could not be identified and may cause a bias in the interpretation of this study.

Our study has some other limitations that should be acknowledged. First, there is an inherent selection bias because of the retrospective design. Second, the descriptive study lacked important information regarding the indication of admission into the hospital and the ICU. The unrecognized indication of surgery type performed on the patients hindered subsequent evaluation of the effects of any particular treatment intervention. We could only assume that there was uniform assessment and management of patients by the surgeons even under the implement of health policy such as diagnosis-related group (DRG) since 2010 in Taiwan. Third, because the total charge for hip fracture is estimated to be three times that of hospital care [11], the study’s assessment of the in-hospital charges may not reflect the total charge and burden of care for treating high- or medium-risk patients. Furthermore, the charge of the metal plates and screws used at the patient’s own expense were not included in the charge of surgery, and this may result in some bias; however, because such metal plates and screws are expensive and more commonly used in low-risk patients with less severe osteoporotic fracture, the inclusion of such instruments may increase the discrepancy between the charge incurred by low-risk patients and that incurred by high- or medium-risk patients.

## 5. Conclusions

This study of hospitalized trauma patients with femoral fracture according to the OSTA osteoporosis risk classification revealed that high- and medium-risk patients were significantly injured in fall accidents more than low-risk patients were, and had a significantly lower ISS, shorter hospital LOS, and less total expenditure. However, shorter hospital LOS and less total expenditure were not found on comparing well-paired, propensity score-matched patients according to covariates such as mechanism, GCS, injuries based on AIS, and ISS, indicating that the difference may be attributed to the patients’ associated injury severity. In this study, we found the surgery charges to be significantly lower in high-risk and medium-risk patients than in low-risk patients, which may be explained by a less aggressive surgery performed by orthopedists on these older patients with a higher risk of osteoporosis.

## Figures and Tables

**Table 1:** Demographics and injury characteristics of high-risk (OSTA < −4), medium-risk (−1 ≥ OSTA ≥ −4), and low-risk (OSTA > −1) patients with femoral fracture.

Variables	High-Risk OSTA < −4 *n* = 814 (I)	Medium-Risk −1 ≥ OSTA ≥ −4 *n* = 634 (II)	Low-Risk OSTA > −1 *n* = 638 (III)	OR (95% CI) I vs. III	*p*	OR (95% CI) II vs. III	*p*
**Age**	82.3 ± 6.2	74.0 ± 7.6	59.4 ± 10.8	-	<0.001	-	<0.001
**Gender**					<0.001		<0.001
**Male**	207 (25.4)	215 (33.9)	370 (58.0)	0.2 (0.20–0.31)		0.4 (0.30–0.47)	
**Female**	607 (74.6)	419 (66.1)	268 (42.0)	4.0 (3.24–5.06)		2.7 (2.14–3.38)	
**Co-morbidity**							
**DM**	192 (23.6)	243 (38.3)	179 (28.1)	0.8 (0.63–1.00)	0.053	1.6 (1.26–2.02)	<0.001
**HTN**	489 (60.1)	384 (60.6)	265 (41.5)	2.1 (1.72–2.62)	<0.001	2.2 (1.73–2.71)	<0.001
**CAD**	81 (10.0)	65 (10.3)	40 (6.3)	1.7 (1.11–2.45)	0.012	1.7 (1.13–2.57)	0.010
**CHF**	24 (2.9)	17 (2.7)	6 (0.9)	3.2 (1.30–7.88)	0.008	2.9 (1.14–7.41)	0.020
**CVA**	100 (12.3)	88 (13.9)	62 (9.7)	1.3 (0.93–1.82)	0.123	1.5 (1.06–2.12)	0.021
**Mechanism, *n* (%)**							
**Motor vehicle**	1 (0.1)	3 (0.5)	19 (3.0)	0.0 (0.01–0.30)	<0.001	0.2 (0.05–0.53)	0.001
**Motorcycle**	39 (4.8)	99 (15.6)	218 (34.2)	0.1 (0.07–0.14)	<0.001	0.4 (0.27–0.47)	<0.001
**Bicycle**	23 (2.8)	43 (6.8)	33 (5.2)	0.5 (0.31–0.92)	0.021	1.3 (0.84–2.13)	0.226
**Pedestrian**	11 (1.4)	8 (1.3)	5 (0.8)	1.7 (0.60–5.02)	0.304	1.6 (0.53–4.97)	0.397
**Fall**	733 (90.0)	474 (74.8)	344 (53.9)	7.7 (5.86–10.21)	<0.001	2.5 (2.00–3.21)	<0.001
**Struck by/against**	7 (0.9)	7 (1.1)	18 (2.8)	0.3 (0.12–0.72)	0.004	0.4 (0.16–0.93)	0.027
**BAC > 50 mg/dL, *n* (%)**	1 (0.1)	3 (0.5)	34 (5.3)	0.0 (0.00–0.16)	<0.001	0.1 (0.03–0.28)	<0.001
**GCS**	14.7 ± 1.1	14.8 ± 1.0	14.7 ± 1.6	—	0.339	—	0.057
**AIS ≥ 3, *n* (%)**							
**Head/Neck**	42 (5.2)	29 (4.6)	72 (11.3)	0.4 (0.29–0.64)	<0.001	0.4 (0.24–0.59)	<0.001
**Face**	9 (1.1)	9 (1.4)	39 (6.1)	0.2 (0.08–0.36)	<0.001	0.2 (0.11–0.46)	<0.001
**Thorax**	19 (2.3)	16 (2.5)	50 (7.8)	0.3 (0.16–0.48)	<0.001	0.3 (0.17–0.54)	<0.001
**Abdomen**	14 (1.7)	7 (1.1)	23 (3.6)	0.5 (0.24–0.92)	0.024	0.3 (0.13–0.70)	0.003
**Extremity**	810 (99.5)	633 (99.8)	637 (99.8)	0.3 (0.04–2.85)	0.392	1.0 (0.06–15.92)	1.000
**ISS, median (IQR)**	9 (9–9)	9 (9–9)	9 (9–9)	-	<0.001	-	<0.001
**＜16**	797 (97.9)	622 (98.1)	583 (91.4)	4.4 (2.54–7.70)	<0.001	4.9 (2.59–9.22)	<0.001
**16–24**	9 (1.1)	4 (0.6)	28 (4.4)	0.2 (0.11–0.52)	<0.001	0.1 (0.05–0.40)	<0.001
**≥25**	8 (1.0)	8 (1.3)	27 (4.2)	0.2 (0.10–0.50)	<0.001	0.3 (0.13–0.64)	0.001
**Mortality, *n* (%)**	9 (1.1)	5 (0.8)	9 (1.4)	0.8 (0.31–1.98)	0.602	0.6 (0.19–1.67)	0.288
**LOS in hospital (days)**	9.9 ± 7.0	9.9 ± 7.4	11.1 ± 9.6	-	0.010	-	0.016
**ICU admission, *n* (%)**	72 (8.8)	42 (6.6)	64 (10.0)	0.9 (0.61–1.24)	0.441	0.6 (0.42–0.96)	0.028
**LOS in ICU (days)**	7.2 ± 10.4	5.4 ± 5.1	8.6 ± 8.4	—	0.398	—	0.016
**Medical expenses**	3495 ± 2813	3654 ± 3195	4451 ± 5014	—	<0.001	—	0.001
**Charge of operation**	539 ± 209	585 ± 343	758 ± 655	—	<0.001	—	<0.001
**Charge of examination**	228 ± 347	195 ± 297	217 ± 371	—	0.552	—	0.239
**Charge of pharmaceutical**	173 ± 380	155 ± 290	232 ± 721	—	0.063	—	0.013

AIS = abbreviated injury scale; BAC = blood alcohol concentration; CAD = coronary artery disease; CI = confidence interval; CVA = cerebral vascular accident; DM = diabetes mellitus; GCS = Glasgow Coma Scale; HTN = hypertension; ICU = intensive care unit; IQR = interquartile range; ISS = injury severity score; LOS = length of stay; OR = odds ratio.

**Table 2:** Femoral fracture sites in high-risk (OSTA < −4), medium-risk (−1 ≥ OSTA ≥ −4), and low-risk (OSTA > −1) patients.

Femur Fracture Sites	High-Risk OSTA < −4 *n* = 827 (I)	Medium-Risk −1 ≥ OSTA ≥ −4 *n* = 649 (II)	Low-Risk OSTA > −1 *n* = 671 (III)	OR (95% CI) I vs. III	*p*	OR (95% CI) II vs. III	*p*
Proximal-Type A (trochanter)	384 (46.4)	248 (38.2)	197 (29.4)	2.1 (1.68–2.59)	<0.001	1.5 (1.18–1.87)	0.001
Proximal-Type B (neck)	373 (45.1)	288 (44.4)	218 (32.5)	1.7 (1.38–2.11)	<0.001	1.7 (1.33–2.07)	<0.001
Proximal-Type C (head)	2 (0.2)	1 (0.2)	12 (1.8)	0.1 (0.03–0.60)	0.002	0.1 (0.01–0.65)	0.003
Shaft	29 (3.5)	59 (9.1)	127 (18.9)	0.2 (0.10–0.24)	<0.001	0.4 (0.31–0.60)	<0.001
Distal	39 (4.7)	53 (8.2)	117 (17.4)	0.2 (0.16–0.34)	<0.001	0.4 (0.30–0.59)	<0.001

CI = confidence interval; OR = odds ratio; *n* = fracture sites.

**Table 3:** Covariates of high-risk (OSTA < −4) and low-risk (OSTA > −1) patients before and after propensity score matching (1:1 matching via Greedy method).

Variables	Before Matching				After Matching			
	High-Risk OSTA < −4 *n* = 814	Low-Risk OSTA > −1 *n* = 638	OR (95% CI)	*p*	High-Risk OSTA < −4 *n* = 408	Low-Risk OSTA > −1 *n* = 408	OR (95% CI)	*p*
**Mechanism, *n* (%)**								
**Motor vehicle**	1 (0.1)	19 (3.0)	0.0 (0.01–0.30)	<0.001	1 (0.2)	1 (0.2)	1.0 (0.06–16.04)	1.000
**Motorcycle**	39 (4.8)	218 (34.2)	0.1 (0.07–0.14)	<0.001	38 (9.3)	38 (9.3)	1.0 (0.62–1.60)	1.000
**Bicycle**	23 (2.8)	33 (5.2)	0.5 (0.31–0.92)	0.021	22 (5.4)	22(5.4)	1.0 (0.55–1.84)	1.000
**Pedestrian**	11 (1.4)	5 (0.8)	1.7 (0.60–5.02)	0.304	4 (1.0)	4 (1.0)	1.0 (0.25–4.03)	1.000
**Fall**	733 (90.0)	344 (53.9)	7.7 (5.86–10.21)	<0.001	338 (82.8)	338 (82.8)	1.0 (0.70–1.44)	1.000
**Struck by/against**	7 (0.9)	18 (2.8)	0.3 (0.12–0.72)	0.004	5 (1.2)	5 (1.2)	1.0 (0.29–3.48)	1.000
**GCS**	14.7 ± 1.1	14.7 ± 1.6	—	0.339	14.9 ± 0.8	14.9 ± 0.7	—	0.781
**AIS ≥ 3, *n* (%)**								
**Head/Neck**	42 (5.2)	72 (11.3)	0.4 (0.29–0.64)	<0.001	18 (4.4)	18 (4.4)	1.0 (0.51–1.95)	1.000
**Face**	9 (1.1)	39 (6.1)	0.2 (0.08–0.36)	<0.001	7 (1.7)	7 (1.7)	1.0 (0.35–2.88)	1.000
**Thorax**	19 (2.3)	50 (7.8)	0.3 (0.16–0.48)	<0.001	11 (2.7)	11 (2.7)	1.0 (0.43–2.33)	1.000
**Abdomen**	14 (1.7)	23 (3.6)	0.5 (0.24–0.92)	0.024	5 (1.2)	5 (1.2)	1.0 (0.29–3.48)	1.000
**Extremity**	810 (99.5)	637 (99.8)	0.3 (0.04–2.85)	0.392	408 (100.0)	408 (100.0)	—	—
**ISS, median (IQR)**	9 (9–9)	9 (9–9)	—	<0.001	9 (9–9)	9 (9–9)	—	0.774
**LOS in hospital (days)**	9.9 ± 7.0	11.1 ± 9.6	—	0.010	9.7 ± 6.7	9.6 ± 7.7	—	0.832
**ICU admission, *n* (%)**	72 (8.8)	64 (10.0)	0.9 (0.61–1.24)	0.441	31 (7.6)	19 (4.7)	1.7 (0.94–3.03)	0.080
**Medical expenses**	3495 ± 2813	4451 ± 5014	—	<0.001	3517 ± 2793	3733 ± 3539	—	0.332
**Charge of operation**	539 ± 209	758 ± 655	—	<0.001	551 ± 200	657 ± 489	—	<0.001
**Charge of examination**	228 ± 347	217 ± 371	—	0.552	220 ± 352	202 ± 373	—	0.485
**Charge of pharmaceutical**	173 ± 380	232 ± 721	—	0.063	181 ± 425	171 ± 475	—	0.754

AIS = abbreviated injury scale; CI = confidence interval; GCS = Glasgow Coma Scale; ICU = intensive care unit; IQR = interquartile range; ISS = injury severity score; LOS = length of stay; OR = odds ratio.

**Table 4:** Covariates of medium-risk (−1 ≥ OSTA ≥ −4) and low-risk (OSTA > −1) patients before and after propensity score matching (1:1 matching via Greedy method).

Variables	Before Matching				After Matching			
	Medium-Risk −1 ≥ OSTA ≥ −4 *n* = 634	Low-Risk OSTA > −1 *n* = 638	OR (95% CI)	*p*	Medium-Risk −1 ≥ OSTA ≥ −4 *n* = 477	Low-Risk OSTA > −1 *n* = 477	OR (95% CI)	*p*
**Mechanism, *n* (%)**								
**Motor vehicle**	3 (0.5)	19 (3.0)	0.2 (0.05–0.53)	0.001	3 (0.6)	3 (0.6)	1.0 (0.20–4.98)	1.000
**Motorcycle**	99 (15.6)	218 (34.2)	0.4 (0.27–0.47)	<0.001	99 (20.8)	99 (20.8)	1.0 (0.73–1.37)	1.000
**Bicycle**	43 (6.8)	33 (5.2)	1.3 (0.84–2.13)	0.226	33 (6.9)	33 (6.9)	1.0 (0.61–1.65)	1.000
**Pedestrian**	8 (1.3)	5 (0.8)	1.6 (0.53–4.97)	0.397	2 (0.4)	2 (0.4)	1.0 (0.14–7.13)	1.000
**Fall**	474 (74.8)	344 (53.9)	2.5 (2.00–3.21)	<0.001	333 (69.8)	333 (69.8)	1.0 (0.76–1.32)	1.000
**Struck by/against**	7 (1.1)	18 (2.8)	0.4 (0.16–0.93)	0.027	7 (1.5)	7 (1.5)	1.0 (0.35–2.87)	1.000
**GCS**	14.8 ± 1.0	14.7 ± 1.6	—	0.057	14.9 ± 0.8	14.9 ± 0.9	—	1.000
**AIS ≥ 3, *n* (%)**								
**Head/Neck**	29 (4.6)	72 (11.3)	0.4 (0.24–0.59)	<0.001	22 (4.6)	22 (4.6)	1.0 (0.55–1.83)	1.000
**Face**	9 (1.4)	39 (6.1)	0.2 (0.11–0.46)	<0.001	8 (1.7)	8 (1.7)	1.0 (0.37–2.69)	1.000
**Thorax**	16 (2.5)	50 (7.8)	0.3 (0.17–0.54)	<0.001	14 (2.9)	14 (2.9)	1.0 (0.47–2.12)	1.000
**Abdomen**	7 (1.1)	23 (3.6)	0.3 (0.13–0.70)	0.003	5 (1.0)	5 (1.0)	1.0 (0.29–3.48)	1.000
**Extremity**	633 (99.8)	637 (99.8)	1.0 (0.06–15.92)	1.000	477 (100.0)	477 (100.0)	—	—
**ISS, median (IQR)**	9 (9–9)	9 (9–9)	—	<0.001	9 (9–9)	9 (9–9)	—	0.947
**LOS in hospital (days)**	9.9 ± 7.4	11.1 ± 9.6	—	0.016	10.1 ± 7.6	9.7 ± 8.2	—	0.406
**ICU admission, *n* (%)**	42 (6.6)	64 (10.0)	0.6 (0.42–0.96)	0.028	33 (6.9)	27 (5.7)	1.2 (0.73–2.10)	0.424
**Medical expenses**	3654 ± 3195	4451 ± 5014		0.001	3729 ± 3367	3869 ± 4402	—	0.581
**Charge of operation**	585 ± 343	758 ± 655	—	<0.001	595 ± 366	671 ± 510	—	0.008
**Charge of examination**	195 ± 297	217 ± 371	—	0.239	202 ± 313	194 ± 361	—	0.703
**Charge of pharmaceutical**	155 ± 290	232 ± 721	—	0.013	159 ± 305	165 ± 454	—	0.793

AIS = abbreviated injury scale; CI = confidence interval; GCS = Glasgow Coma Scale; ICU = intensive care unit; IQR = interquartile range; ISS = injury severity score; LOS = length of stay; OR = odds ratio.

**Table 5:** Covariates of high-risk (OSTA < −4) and medium-risk (−1 ≥ OSTA ≥ −4) patients before and after propensity score matching (1:1 matching via Greedy method).

Variables	Before Matching				After Matching			
	High-Risk OSTA < −4 *n* = 814	Medium-Risk −1 ≥ OSTA ≥ −4 *n* = 634	OR (95% CI)	*p*	High-Risk OSTA < −4 *n* = 545	Medium-Risk −1 ≥ OSTA ≥ −4 *n* = 545	OR (95% CI)	*p*
**Mechanism, *n* (%)**								
**Motor vehicle**	1 (0.1)	3 (0.5)	0.3 (0.03–2.49)	0.325	1 (0.2)	1 (0.2)	1.0 (0.06–16.03)	1.000
**Motorcycle**	39 (4.8)	99 (15.6)	0.3 (0.19–0.40)	<0.001	37 (6.8)	37 (6.8)	1.0 (0.62–1.60)	1.000
**Bicycle**	23 (2.8)	43 (6.8)	0.4 (0.24–0.67)	<0.001	22 (4.0)	22 (4.0)	1.0 (0.55–1.83)	1.000
**Pedestrian**	11 (1.4)	8 (1.3)	1.1 (0.43–2.68)	0.882	7 (1.3)	7 (1.3)	1.0 (0.35–2.87)	1.000
**Fall**	733 (90.0)	474 (74.8)	3.1 (2.28–4.09)	<0.001	474 (87.0)	474 (87.0)	1.0 (0.70–1.42)	1.000
**Struck by/against**	7 (0.9)	7 (1.1)	0.8 (0.27–2.23)	0.638	4 (0.7)	4 (0.7)	1.0 (0.25–4.02)	1.000
**GCS**	14.7 ± 1.1	14.8 ± 1.0	—	0.190	14.8 ± 0.8	14.8 ± 0.8	—	0.819
**AIS ≥ 3, *n* (%)**								
**Head/Neck**	42 (5.2)	29(4.6)	1.1 (0.70–1.84)	0.609	18 (3.3)	18 (3.3)	1.0 (0.52–1.94)	1.000
**Face**	9 (1.1)	9 (1.4)	0.8 (0.31–1.97)	0.593	4 (0.7)	4 (0.7)	1.0 (0.25–4.02)	1.000
**Thorax**	19 (2.3)	16 (2.5)	0.9 (0.47–1.81)	0.816	9 (1.7)	9 (1.7)	1.0 (0.39–2.54)	1.000
**Abdomen**	14 (1.7)	7 (1.1)	1.6 (0.63–3.91)	0.331	4 (0.7)	4 (0.7)	1.0 (0.25–4.02)	1.000
**Extremity**	810 (99.5)	633 (99.8)	0.3 (0.04–2.87)	0.393	544 (99.8)	544 (99.8)	1.0 (0.06–16.03)	1.000
**ISS, median (IQR)**	9 (9–9)	9 (9–9)	—	0.435	9 (9–9)	9 (9–9)	—	0.722
**LOS in hospital (days)**	9.9 ± 7.0	9.9 ± 7.4	—	0.966	9.7 ± 6.6	9.5 ± 6.7	—	0.705
**ICU admission, *n* (%)**	72 (8.8)	42 (6.6)	1.4 (0.92–2.03)	0.120	44 (8.1)	33 (6.1)	1.4 (0.85–2.18)	0.193
**Medical expenses**	3495 ± 2813	3654 ± 3195	—	0.315	3472 ± 2638	3465 ± 2821	—	0.968
**Charge of operation**	539 ± 209	585 ± 343	—	0.003	554 ± 204	555 ± 253	—	0.959
**Charge of examination**	228 ± 347	195 ± 297	—	0.053	212 ± 333	193 ± 303	—	0.305
**Charge of pharmaceutical**	173 ± 380	155 ± 290	—	0.326	163 ± 333	146 ± 272	—	0.345
